# Two dimensional blue native/SDS-PAGE to identify mitochondrial complex I subunits modified by 4-hydroxynonenal (HNE)

**DOI:** 10.3389/fphys.2015.00098

**Published:** 2015-03-26

**Authors:** Jinzi Wu, Xiaoting Luo, Liang-Jun Yan

**Affiliations:** ^1^Department of Pharmaceutical Sciences, UNT System College of Pharmacy, University of North Texas Health Science CenterFort Worth, TX, USA; ^2^Department of Biochemistry and Molecular Biology, Gannan Medical UniversityGanzhou, China

**Keywords:** blue native/SDS-PAGE, diabetes, 4-hydroxynonenal, mitochondria, reactive oxygen species, streptozotocin

## Abstract

The lipid peroxidation product 4-hydroxynonenal (HNE) can form protein-linked HNE adducts, thereby impacting protein structure and function. Mitochondrial complex I (NADH-ubiquinone oxidoreductase), containing at least 45 subunits in mammalian cells, sits in a lipid-rich environment and is thus very susceptible to HNE modifications. In this paper, a procedure for the identification of HNE-modified complex I subunits is described. Complex I was isolated by first dimensional non-gradient blue native polyacrylamide gel electrophoresis (BN-PAGE). The isolated complex I band, visualized by either Coomassie blue staining or silver staining, was further analyzed by second dimensional sodium dodecyl sulfate-polyacrylamide gel electrophoresis (SDS-PAGE). HNE-modified proteins were visualized by Western blotting probed with anti-HNE antibodies. HNE-positive bands were then excised and the proteins contained in them were identified by mass spectrometric peptide sequencing. The method was successfully applied for the identification of two complex I subunits that showed enhanced HNE-modifications in diabetic kidney mitochondria.

## Introduction

Lipids are targets of oxidative damage induced by reactive oxygen species (ROS). 4-hydroxynonenal (HNE) is a lipid peroxidation byproduct derived from membrane lipid oxidation by ROS (Dalleau et al., 2013). It is a signaling molecule (Vatsyayan et al., 2011) and can react with certain protein amino acid residues such as lysine, histidine, and cysteine (Uchida and Stadtman, 1992a,b), leading to changes in protein structure and function. Indeed, HNE and HNE -modified proteins have been suggested to be involved in the pathogenesis of many age-related diseases such as Alzheimer's (Gwon et al., 2012) and Parkinson's disease (Farooqui and Farooqui, 2011), cardiovascular disease (Anderson et al., 2012), diabetes (Cohen et al., 2013), and cancer (Warnakulasuriya et al., 2008). Therefore, studying HNE modified proteins may help understand the mechanisms of cell death in a given pathophysiological condition.

Mitochondrial complex I (NADH-ubiquinone oxidoreductase) is the first complex in the mitochondrial electron transport chain (Vinothkumar et al., 2014). It takes electrons from nicotinamide adenine dinucleotide (NADH) and passes them to coenzyme Q (Vinothkumar et al., 2014). During this NADH oxidation process, protons are pumped via complex I into the intermembrane space, forming a proton gradient across the inner membrane that drives the synthesis of ATP. In the meantime, NADH oxidation also drives complex I production of superoxide anion (Hirst et al., 2008; Treberg et al., 2011), the precursor of other ROS such as hydrogen peroxide, hydroxyl radical, and peroxynitrite when nitric oxide is available (Yan, 2014). As proteins are susceptible to oxidative damage, complex I is thus both a source and target of ROS. It has been established that complex I dysfunction is linked to numerous aging-related diseases such as Parkinson's disease and diabetes (Cooper et al., 1992; Schapira, 1998; Fassone and Rahman, 2012).

Complex I is a multisubunit complex consisting of at least 45 subunits in mammalian cells (Carroll et al., 2006). Many of the subunits are redox-sensitive proteins that are susceptible to attacks by ROS and lipid peroxidation productions such as HNE (Hattori et al., 1991; Yoritaka et al., 1996; Rafique et al., 2001), which may lead to impairment of complex I function. In this article, we present a gel-based method for isolation and resolution of complex I subunits and identification of HNE-modified complex I proteins. The method involves isolation of whole complex I using first dimensional blue native polyacrylamide gel electrophoresis (BN-PAGE) followed by second dimensional sodium dodecyl sulfate-polyacrylamide gel electrophoresis (SDS-PAGE) to resolve individual complex I subunits. HNE modified proteins are identified by Western blotting analysis using anti-HNE antibodies. Protein bands showing positive HNE immuno-staining are then excised for identification by mass spectrometric peptide sequencing. It should be noted that we took the BN-/SDS-PAGE approach for our study because the conventional IEF/SDS-PAGE would disrupt complex I association and does not yield an intact complex for further in-gel activity measurement and complex I subunit analysis.

## Materials and methods

### Chemicals and reagents

Sucrose and mannitol were purchased from BDH Chemicals and Mallickrodt Chemicals, respectively. Bis-Tris, tricine, and amino-caproic acid were purchased from MB Biochemicals (Irvine, CA). Pre-stained SDS-PAGE markers were purchased from Thermo Scientific (Pittsburgh, PA). Bradford protein assay solution and Coomassie brilliant blue (CBB) R-250 were from Bio-Rad laboratories (Richmond, CA). Silver nitrate, streptozotocin (STZ), sodium citrate, NADH, EDTA, n-dodecyl-β-D-maltoside (DDM), and nitro blue tetrazolium (NBT) chloride tablets were obtained from Sigma (St. Louis, MO, USA). Serva Blue G was purchased from Serva (Heidelberg, Germany). Rabbit anti-HNE polyclonal antibodies (IgG) and goat anti-rabbit IgG conjugated with horseradish peroxidase were purchased from US Biological (Salem, MA) and Invitrogen (San Diego, CA), respectively. Hybond-C membrane and a Western blot detection kit were obtained from GE Healthcare (Piscataway, NJ).

### Animals and induction of diabetes

Young adult male Sprague Dawley rats (2–6 months old) purchased from Charles River were used in this study. Diabetes was induced by a single intraperitoneal injection of STZ (60 mg/kg body weight) after overnight fasting (Gajdosik et al., 1999). STZ was prepared fresh by dissolving in 0.1 M citrate buffer pH 4.5 and control animals received citrate buffer only. Blood glucose levels were monitored once a week using blood glucose test strips (FreeStyle lite from Abbott Diabetes Care Inc., Alameda, California). Animals with blood glucose levels exceeding 200 mg/dl were deemed to be diabetic. Four weeks after STZ injections, animals were sacrificed and tissues were collected.

### Isolation of tissue mitochondria

Mitochondria from either heart or kidney were used in this study. The procedures for mitochondria preparations from both tissues were essentially the same as previously described (Navarro et al., 2004). Briefly, tissues were homogenized (1 g tissue per 10 ml isolation buffer) in mitochondrial isolation buffer containing 70 mM sucrose, 230 mM mannitol, 15 mM MOPS (pH 7.2), and 1 mM potassium EDTA. The homogenates were then centrifuged at 800 g for 10 min at 4°C. The supernatant was kept and further centrifuged at 8000 g also for 10 min at 4°C. The resulting pellet, containing mitochondria, was washed once with 10 ml isolation buffer and centrifuged again under the same conditions. The obtained mitochondrial pellet was either stored at -80°C or used immediately. For preparation of mitochondrial membrane proteins, mitochondrial pellet was resuspended in 30 mM potassium phosphate and sonicated four times 30 s with 1 min interval. The resuspension was then centrifuged at 80,000 × g for 30 min and the resulting pellet contained mitochondrial membranes (Yan et al., 1997).

### First dimensional blue native polyacrylamide gel electrophoresis (BN-PAGE) and in-gel complex I activity staining

A non-gradient BN-PAGE was employed as previously described (Yan and Forster, 2009). Mitochondrial pellet was solubilized in a BN-PAGE sample buffer containing 0.75 M aminocaproic acid, 75 mM Bis-Tris, and 1% DDM, pH 7.0. After brief sonication and further incubation on ice for 60 min, the solution was centrifuged at 8000 g and the resulting supernatant was used for BN-PAGE. Protein concentrations were determined by the Bradford assay (Bradford, 1976). Gel buffer contained 500 mM aminocaproic acid, 50 mM Bis-Tris. The cathode buffer contained 50 mM tricine, 15 mM Bis-Tris pH 7.0 with or without 0.02% CBB. The anode buffer contained 50 mM Bis-Tris, pH 7.0. Gel was run at 150 voltages using CBB-containing cathode buffer until the front reached at one-third of the gel where the cathode buffer was replaced with the one that didn't have CBB. Gel running was resumed at 200 voltages until complete. After gel electrophoresis, the gel was stained either by CBB staining or by complex I activity staining. Activity staining was achieved at room temperature by incubating the gels or gel strips in 50 mM potassium phosphate buffer (pH 7.0) containing 0.1 mg/ml NADH and 0.2 mg/ml NBT (Yan and Forster, 2009).

### Second dimensional SDS-PAGE and western blotting

For second dimensional SDS-PAGE, a 10% resolving gel was usually performed. Gel strips derived from the first dimensional blue native gel was equilibrated in 5% 2-mercaptoethanol, 62.5 mM Tris-HCl (pH 6.8), 2% SDS, and 10 mM glycerol for 20 min (Yan et al., 1998). The strip or complex I band was then placed onto the second dimensional gel for electrophoresis as previously described (Yan et al., 2007). Usually two gels were run simultaneously. One gel was for CBB or silver staining, and the other gel was used for membrane transfer and Western blotting according to standard procedures. HNE signals were revealed by anti-HNE antibodies. All densitometric quantifications were performed using AlphaEaseFC image analysis software (Alpha Innotech, SanLeandro, CA). Statistical analysis was performed using GraphPad software. ^*^ *P* < 0.05 indicates significant differences between control and STZ groups.

### Silver staining

A procedure of silver staining (Yan et al., 2000) was modified and used in this study. Following electrophoresis, gels were fixed in 50% methanol, 12% acetic acid containing 0.05% formalin for 1 h. The gels were then washed with double-distilled (dd) water for three times with each time gently shaking for 5 min. This was followed by sensitization in 0.02% sodium thiosulfate for 2 min. The gels were again washed with dd water for three times with each time also gently shaking for 5 min. Silver staining was performed by incubating the gels in a silver nitrate (0.2%) solution for 20 min. After washing twice in water for 1 min each, gel bands were developed in an ice-cold 6% sodium bicarbonate. Staining was stopped by a solution containing 50% methanol and 12% acetic acid. The gels were kept in a solution containing 10% methanol and 8% acetic acid. This method is compatible with mass spectrometric peptide sequencing (Yan et al., 2000).

### Mass spectrometric peptide sequencing

Protein identification via mass spectrometric peptide sequencing was conducted at ProtTech (Phoneixville, PA) using NanoLC-MS/MS peptide sequencing technology. Briefly, the silver-stained gel band was excised, cleaned, and digested with sequencing grade trypsin. The resulting protein mixture was analyzed by an LC-MS/MS equipment. The collected mass spectrometric data were used to search the protein database using ProtTech's software suite.

## Results and discussion

### First dimensional BN-PAGE

**Figure 1:**
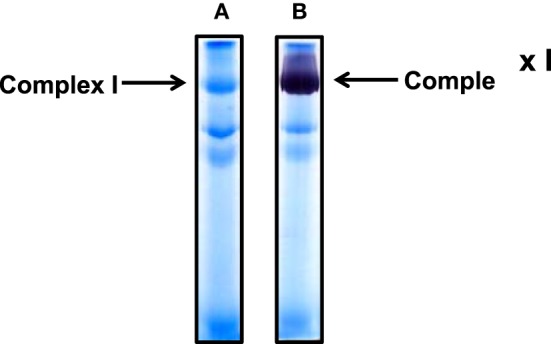
**Non-gradient blue native gel electrophoresis of cardiac mitochondria isolated from rat heart. (A)** Gel image after electrophoresis without further CBB staining; **(B)** complex I activity staining by NADH and NBT. An 8% resolving gel was performed.

**Figure 2:**
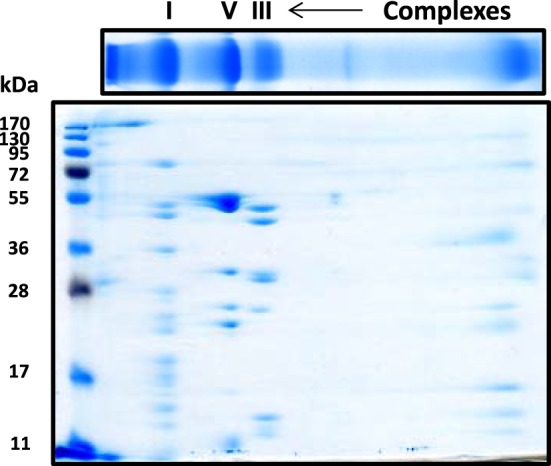
**Second dimensional SDS-PAGE of complex I subunits together with those of complex V and complex III**. The blue native gel strip was placed on top of the second dimensional SDS gel (10% resolving). Gel was stained by CBB staining.

**Figure 3:**
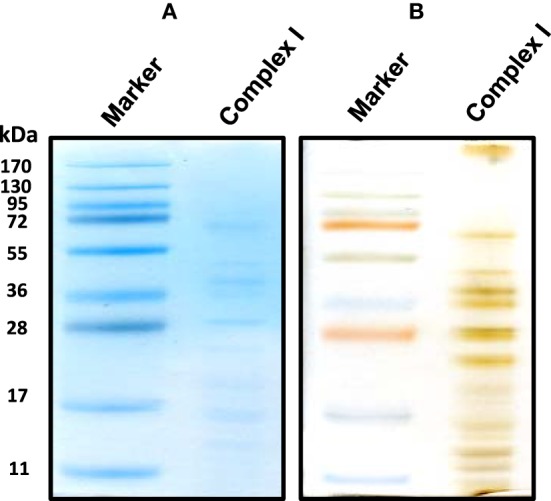
**An alternative second dimensional SDS-PAGE approach whereby the complex I band derived from the gels shown in Figure 1 was placed on top of the second dimensional SDS gel**. Both CBB staining **(A)** and silver staining **(B)** of complex I proteins bands were shown.

Cardiac mitochondria were used in our condition set-up experiments (Figures 1–3). For complex I isolation, we used a non-gradient blue native PAGE method as previously described (Yan and Forster, 2009). Figure 1A shows complex I band localization after gel electrophoresis. It was not necessary to further stain the gel after electrophoresis as complex I was always visible due to its pre-binding of Coomassie blue achieved during sample preparation. Figure 1B shows in-gel complex I activity staining by incubating the gels strips in a solution containing NADH and NBT.

### Second dimensional SDS-PAGE

For the resolution of each individual complex I subunit, we initially took two approaches. The first one was to turn the whole gel strip 90 degrees counter clockwise and layered it onto the second dimensional gel. As shown in Figure 2, this approach separated not only complex I subunits, but also those of complexes V and III, among others. The second approach was to excise the complex I band from the blue native gel, and placed this band onto a second dimensional gel, resulting in the resolution of only complex I subunits as shown in Figure 3 whereby both CBB staining and silver staining of individual complex I proteins were demonstrated. This approach was preferred because it would facilitate band matching and excision between stained gels and Western blot membranes as no other complexes were present.

### Western blot and identification of complex i subunits modified by HNE

**Figure 4:**
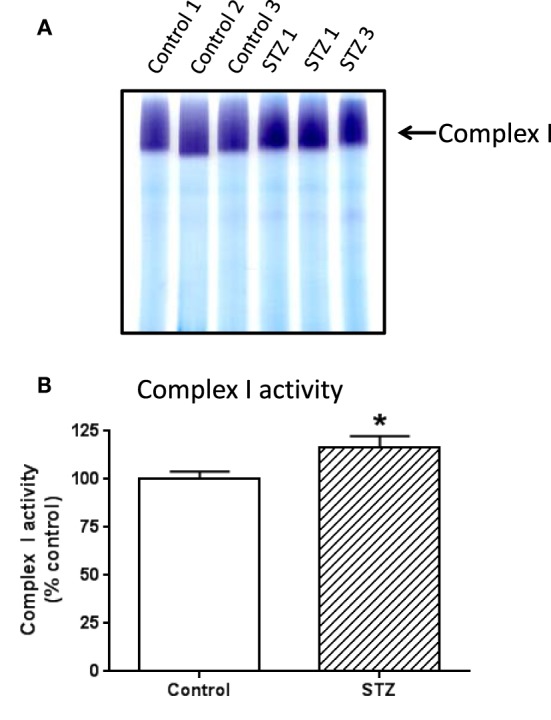
**In-gel complex I activity staining and quantitation of complex I activity in each group of rats**. Shown was comparison between control and diabetic (STZ) mitochondria isolated from rat kidney; **(A)** BN-PAGE activity staining, **(B)** densitometric quantification of complex I activity based on **(A)**. *P* < 0.05 indicates significant difference between control and STZ groups.

**Figure 5:**
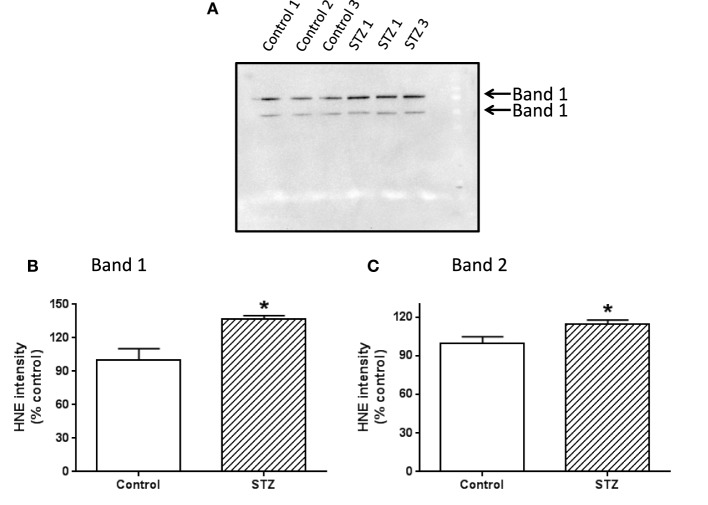
**Western blot detection of HNE modification of complex I subunits**. Two bands showing HNE modification were indicated in **(A)**. **(B,C)** Show the densitometric quantification of HNE content in bands 1 and 2, respectively. *P* < 0.05 indicates significant difference between control and STZ groups.

**Figure 6:**
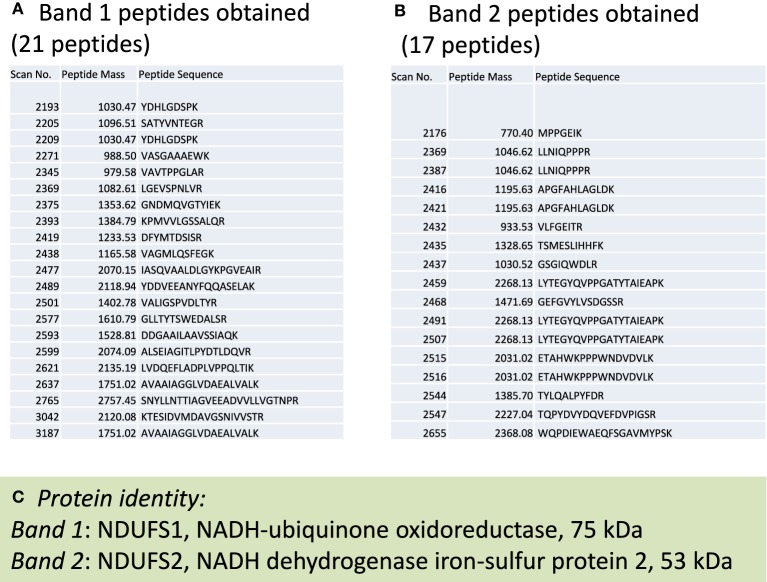
**Mass spectrometric peptide sequencing of band 1 and band 2**. Shown are **(A)** 21 peptides that matched to NDUFS1, **(B)** 17 peptides that matched NDUFS2, and **(C)** the identity of each protein indicated in Figure 5A.

To investigate which complex I subunits could undergo HNE modifications, we then switched to kidney mitochondria isolated from STZ diabetic rats. This switch was due to our observation that there were no detectable changes in cardiac complex I activity in STZ diabetic rats 4 weeks post STZ injection. The idea was to explore which subunits underwent enhanced HNE modifications under diabetic conditions as it is possible that kidney mitochondrial proteins may exhibit enhanced HNE modifications in diabetes (Sivitz and Yorek, 2010). Results are shown in Figures 4, 5. Figure 4A shows a first dimensional in-gel complex I activity staining between control and diabetes. Figure 4B shows densitometric quantification of complex I activity in each group whereby complex I activity in STZ diabetes was higher than that in the control group. Figure 5A shows anti-HNE immunostaining of complex I subunits, in which two prominent bands could be visualized to show a basal level of HNE modification that was enhanced by STZ-induced diabetes. HNE content in each band was also higher in STZ diabetes group than in control group (Figures 5B,C). These two bands were excised and subjected to mass spectrometric peptide sequencing. As shown in Figure 6, there were 21 peptides in band 1 (Figure 6A) that matched to the NADH-ubiquinone oxidoreductase 75 kDa subunit (NDUFS1) and 17 peptides in band 2 (Figure 6B) that matched to the NADH dehydrogenase iron–sulfur protein 2 (NDUFS2). Hence, the two proteins identified were NDUFS1 (75 kDa) and NDUFS2 (53 kDa), respectively (Figure 6C). Both have been reported to undergo HNE modifications under different experimental conditions (Choksi et al., 2008; Zhao et al., 2014). It should be noted that the reason that these two proteins could be positively identified is likely due to the fact that they are the most abundant subunits in complex I, which is the drawback of all gel-based proteomic approaches for identification of posttranslationally modified proteins.

For the first dimensional blue native gel analysis, complex I from tissue mitochondria are always well-resolved. The band can be clearly observed after gel electrophoresis without further CBB staining. Therefore, either the CBB staining band or the activity staining band can be excised for second dimensional SDS-PAGE followed by Western blotting. However, for cultured cells, we have found that a clear complex I band usually failed to be observed even after further CBB staining because of low complex I levels. In this situation, the existence of a well-resolved complex I band could only be visualized by in-gel activity staining which is more sensitive than that of CBB staining. Therefore, for analysis of complex I from cultured cells, the activity containing band should be excised for further analysis.

It should be noted that when whole mitochondrial preparations are loaded for the isolation of complex I in the first dimensional blue native gel analysis, certain non-complex I proteins can co-migrate with complex I, resulting in contamination of complex I by other proteins. Therefore, the use of mitochondrial membrane preparations that contain all complex I subunits should be preferred for identification of complex I subunits.

It should also be noted that the method described in this article may also be used for the analysis of other types of posttranslational modifications such as acetylation (Fritz et al., 2012) and carbonylation (Yan, 2009). For carbonylation analysis, however, the first dimensional gel strips will need to be incubated with carbonyl probes such as 2,4-dinitrophenylhydrazine or biotin-containing probes (Yan and Forster, 2011) before second dimensional gel electrophoresis.

In summary, we present in this paper a method for identification of complex I subunits that can be modified by the lipid peroxidation product HNE. The procedure involves complex I isolation by BN-PAGE, subunit resolution by SDS-PAGE, Western blot detection of HNE-conjugated proteins using anti-HNE antibodies, and protein identification by mass spectrometric peptide sequencing. The procedure may also find applications in identifying complex I subunits undergoing other types of posttranslational modifications.

### Conflict of interest statement

The authors declare that the research was conducted in the absence of any commercial or financial relationships that could be construed as a potential conflict of interest.

## Abbreviations

CBB: Coomassie brilliant blue
DDM: n-dodecyl-**β**-D-maltoside
HNE: 4-hydroxynonenal
NADH: nicotinamide adenine dinucleotide
LC-MS: liquid chromatography-mass spectrometry
NBT: nitro blue tetrazolium
SDS-PAGE: sodium dodecyl sulfate-polyacrylamide gel electrophoresis
ROS: reactive oxygen species
STZ: streptozotocin.
